# Artificial Intelligence in PET Imaging for Alzheimer’s Disease: A Narrative Review

**DOI:** 10.3390/brainsci15101038

**Published:** 2025-09-25

**Authors:** Andrea Marongiu, Angela Spanu, Barbara Palumbo, Francesco Bianconi, Luca Filippi, Giuseppe Madeddu, Susanna Nuvoli

**Affiliations:** 1 Unit of Nuclear Medicine, Department of Medicine, Surgery and Pharmacy, University of Sassari, Viale San Pietro 8, 07100 Sassari, Italy; amarongiu2@uniss.it (A.M.); aspanu@uniss.it (A.S.); giuseppe.madeddu@email.it (G.M.); 2 Section of Nuclear Medicine and Health Physics, Department of Medicine and Surgery, Università degli Studi di Perugia, Piazza Lucio Severi 1, 06132 Perugia, Italy; barbara.palumbo@unipg.it; 3 Nuclear Medicine Unit, Perugia Hospital, Piazzale G. Menghini 3, 06132 Perugia, Italy; 4 Department of Engineering, Università degli Studi di Perugia, Via Goffredo Duranti 93, 06125 Perugia, Italy; bianco@ieee.org; 5 Department of Biomedicine and Prevention, University of Rome “Tor Vergata”, Via Montpellier 1, 00133 Rome, Italy; luca.filippi@uniroma2.it

**Keywords:** Artificial Intelligence, ^18^F-FDG PET, Amyloid PET, Tau PET, Alzheimer’s disease

## Abstract

The rapid advancements in computer processing, algorithmic development, and the availability of large-scale datasets have positioned Artificial Intelligence (AI) as a valuable tool across multiple domains, including Medicine. In the field of Nuclear Medicine neuroimaging, with Positron Emission Tomography (PET), AI has demonstrated significant potential in improving diagnostic accuracy for neurodegenerative cognitive disorders. This is especially relevant for the early diagnosis, preclinical detection, and prediction of disease progression in Alzheimer’s disease (AD), the most prevalent form of cognitive impairment in individuals over 65 years of age. This narrative review aims to synthesize current advances, explore future directions, and highlight outstanding challenges in the application of Artificial Intelligence to PET imaging for the clinical management of Alzheimer’s disease, with particular focus on three key modalities: ^18^F-FDG PET, Amyloid PET, and Tau PET.

## 1. Introduction

AD is the most common and severe progressive neurodegenerative disorder in the elderly population and, as reported by The World Alzheimer’s Report, by 2050, the number of people affected by AD will exceed 152 million cases with the resulting social and economic relevant implications due to the global aging population [1,2].

In recent years, these findings have prompted intensified research efforts aimed at unraveling the complex pathophysiological mechanisms underlying AD. Furthermore, there has been a change in the clinical approach to AD with an increasingly significant orientation towards the pre-symptomatic phases of the disease [3], such as Mild Cognitive Impairment (MCI) [4] and Subjective Cognitive Decline (SCD) [5].

The acceptance in the medical practice of these preclinical conditions has shifted the focus from the concept of clinical disease to that of a biological entity, so that the term “Alzheimer’s disease” refers to a continuum of neuropathological changes that is therefore defined “in vivo” by biomarkers, rather than by clinical symptoms alone [6]. In this context, early disease detection is becoming more and more crucial. Biological markers and neuroimaging techniques have also been shown to be very helpful in the early characterization of AD and in the assessment of preclinical conditions.

Recently, the NIA-AA criteria included different Nuclear Medicine neuroimaging procedures in the AT(N) framework of AD diagnosis, and three general groups of biomarkers, based on the nature of the pathologic AD process that each expresses, have been identified; A stands for Amyloid aggregates, T for Tau presence, and N for neurodegenerative process [6].

In the label “A”, Amyloid PET is considered an “in vivo” biomarker for brain Amyloid plaques, together with low levels of Ab42 protein in cerebrospinal fluid (CSF). In the label “T”, Tau PET represented an “in vivo” biomarker of fibrillary Tau (P-Tau) with elevated phosphorylated Tau in CSF. In the label “N” ^18^F-FDG PET, measuring brain metabolism, is indicated as the biomarker of neurodegeneration or neuronal injury, with Magnetic Resonance (MR) features and elevated total Tau in CSF [6].

As a consequence, the use of PET or, more recently, of the hybrid technique associated with a low-dose computed tomography (PET/CT), may support earlier risk stratification or patient selection for clinical trials, in order to implement timely interventions and more accurate patient management and allow access to therapies that can modify the course of the disease [7,8].

The use of AI also seems to be a valid tool in AD clinical management. In recent decades, the significant advances in computing power, the rapid development of new algorithms, and the increasing availability of larger and larger amounts of data have contributed to making AI a widely useful tool in numerous human spheres, including the medical field. Although oncology is possibly the hot topic for AI applications now, neurological disorders could also benefit from these developments [9].

In particular, two different techniques, Machine Learning (ML) and Deep Learning (DL), have been widely used, becoming progressively prevalent in the medical context since their use allows for faster development of complex predictive models for clinical decisions that involve a large number of variables [10,11,12,13].

ML is based on the combination of feature engineering + classification models, resulting in algorithms that, previously trained, can identify specific patterns from the data and make use of the results to infer predictions [14].

DL differs from ML in the use of neural networks, larger data requirements, and a reduced human contribution. Whereas ML relies on manually designed (hand-crafted) features, DL automatically learns features directly from the data, therefore bypassing the difficulty of manually gathering and selecting potentially important features [14].

The more common ML procedures in AD neuroimaging are the Supervised (Support Vector Machine, Random Forests, and Logistic Regression), Unsupervised (Clustering, Dimensionality Reduction), and Ensemble Learning algorithms, while the more frequent DL are Convolutional Neural Networks (CNNs), Recurrent Neural Networks (RNNs), and Autoencoders [15].

The rapid and significant increase in scientific interest in the use of AI techniques applied to various neuroimaging modalities, including MR, PET, and PET/CT imaging procedures in Alzheimer’s disease, has resulted in a considerable number of scholarly papers.

To offer a comprehensive perspective of the potential applications of AI in PET imaging for AD, this narrative review provides an overview of the literature and of the different applications of Artificial Intelligence to PET imaging in Alzheimer’s disease, also focusing on key subtopics, ^18^F-FDG PET, Amyloid PET, and Tau PET. The most representative and relevant studies were selected to synthesize current advances, explore future directions, and highlight outstanding challenges in this rapidly evolving field.

## 2. Methodology

Our aim was to produce a narrative review that provides an educational synthesis of representative and original studies on the application of Artificial Intelligence to PET imaging in Alzheimer’s disease. We conducted a PubMed search using the terms “Artificial Intelligence”, “PET”, and “Alzheimer’s disease”, supplemented by modality-specific queries (e.g., “^18^F-FDG PET”, “Amyloid PET”, “Tau PET”), covering the period 2005 through early 2025. Titles and abstracts were screened by two authors (S.N. and A.M.), and full texts of potentially relevant articles were examined. Rather than attempting an exhaustive systematic synthesis or a quantitative meta-analysis, we prioritized studies that the authors judged to be particularly original, representative, or instructive for each thematic area. Consequently, we did not perform a formal reproducibility assessment, a structured risk-of-bias evaluation, or a PRISMA-style workflow. The reader should therefore interpret this manuscript as a curated narrative overview that highlights major advances, illustrative examples, and outstanding challenges in the field.

## 3. Artificial Intelligence, PET Imaging, and Alzheimer’s Disease

Our search retrieved 455 papers published from 2005 to early 2025.

**Figure 1:**
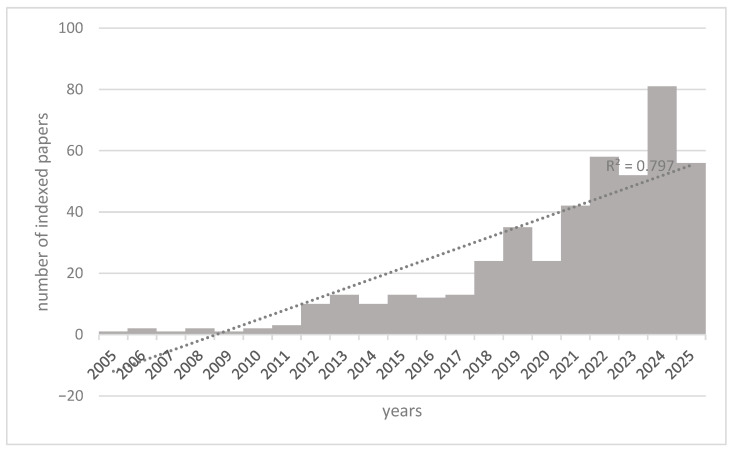
Trend of scientific articles indexed on PubMed concerning Artificial Intelligence, PET, and Alzheimer’s disease, from 2005 to early 2025. The R^2^ value of 0.79 indicated a good fit of the linear model to the data, suggesting that year is a good predictor of the number of outcomes.

As illustrated in Figure 1, there was a near-exponential growth in the number of publications between 2005 and 2024. Moreover, there has been a notable increase in the number of publications starting in 2017, with the highest number of publications occurring in 2024, indicating a notable acceleration in this field of study.

This trend, confirmed by the R^2^ value, could be related to the progressive technological advances both in AI and in PET imaging, as well as to the increased awareness and funding for Alzheimer’s research.

Table 1 summarizes the AI techniques applied to PET or PET/CT images that proved useful in supporting the early diagnosis of Alzheimer’s dementia [15,16], in improving the classification of various AD subtypes [15], and in the prediction of AD progression [15,17].

In particular, ML was considered useful both for early AD diagnosis and for the improvement of AD subtype classification. Ensemble Learning procedures (Bagging, Boosting, Random Forests) were considered for improving the performance of primary classification models [15,18,19,20,21].

The CNNs approach, a DL technique, was employed to aid early AD diagnosis and classification as well as to predict the conversion of Mild Cognitive Impairment to AD. Convolutional Neural Networks and Autoencoders were applied for the evaluation of AD prognosis and in feature extraction [18].

The combined use of ML and DL procedures has proven useful for multimodal data integration [18,22]. These approaches would allow exploiting the different information coming from neuroimaging techniques, such as MR, PET, and PET/CT, as well as from other biomarkers and clinical patients’ data in order to reach better performances and to enhance the accuracy and reliability of the predictive models for AD [15,18,23,24].

### 3.1. Artificial Intelligence, ^18^F-FDG PET and Alzheimer’s Disease

The PubMed search with the keywords “Artificial Intelligence”, “^18^F-FDG PET”, and “Alzheimer’s” disease returned 146 selected items published between 2005 and early 2025.

**Figure 2:**
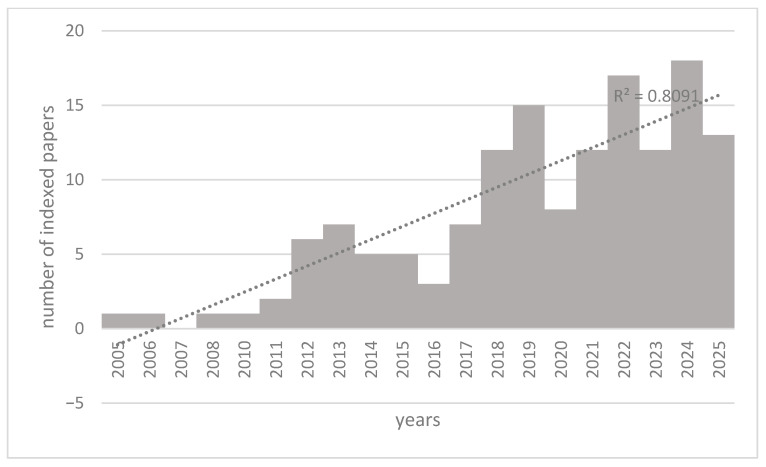
Trend of scientific articles indexed on PubMed from 2005 to early 2025 concerning Artificial Intelligence, ^18^F-FDG PET, and Alzheimer’s disease. The R^2^ value of 0.80 indicated that the linear model fits the data quite well, indicating a strong linear relationship between year and the number of publications.

As illustrated in Figure 2, from 2005 to 2011, the growth of indexed articles was slow and steady, while a turning point occurred around 2012, when the results jumped up, indicating an acceleration in interest.

The interest in these fields showed further growth from 2019 onwards, reaching a peak in 2024. This data could probably be attributed to advances in AI and its application in the medical field, as well as to the growing researchers’ interest in this subject.

Analyzing the linear regression, an R^2^ value of 0.80 suggested that year is a good predictor of the number of publications.

Most researchers have focused their interest on the early diagnosis of AD and on the prediction of the development of MCI into AD in order to provide early treatments.

Three different ML automatic classification methods (Classification tree, Linear Support Vector Machine, and Ridge classifier) were applied to brain ^18^F-FDG PET semi-quantitative data in AD and MCI cases, and, finally, diagnosed with follow-up. The use of ML methods seems to be useful to improve the traditional manual interpretation of ^18^F-FDG PET images. Automatic classification models could indeed identify a specific hypometabolic area in the left temporo-lateral cortical region, involved in AD cases with respect to MCI, thus improving the diagnostic process and the correct diagnostic assessment of cognitive impairments [25].

A relevant prospective study was carried out on a large case series of ^18^F-FDG PET brain images from the Alzheimer’s Disease Neuroimaging Initiative (ADNI), with a final clinical diagnosis of AD and MCI confirmed at follow-up. In these cases, early prediction of Alzheimer’s disease using DL algorithms (CNNs) on ^18^F-FDG brain PET images achieved high specificity and sensitivity (82% and 100%, respectively). These results were obtained approximately 75.8 months prior to the final diagnosis. These data suggested that CNNs offered potential advantages in patient care and therapeutic management, and could be used as an early prediction tool for Alzheimer’s disease, especially in conjunction with other biochemical and imaging tests [26].

The use of DL clustering models on ^18^F-FDG PET images allowed predicting the prognosis of the subtypes for conversion from MCI to AD [27]. Moreover, this model provided useful insights into the heterogeneous pathophysiology of AD, distinguishing specific brain metabolism patterns corresponding to different brain diseases with different clinical profiles [27].

### 3.2. Artificial Intelligence, Amyloid PET, and Alzheimer’s Disease

The PubMed search with the keywords “Artificial Intelligence”, “Amyloid PET”, and “Alzheimer’s disease” returned 193 selected articles, between 2007 and early 2025.

**Figure 3:**
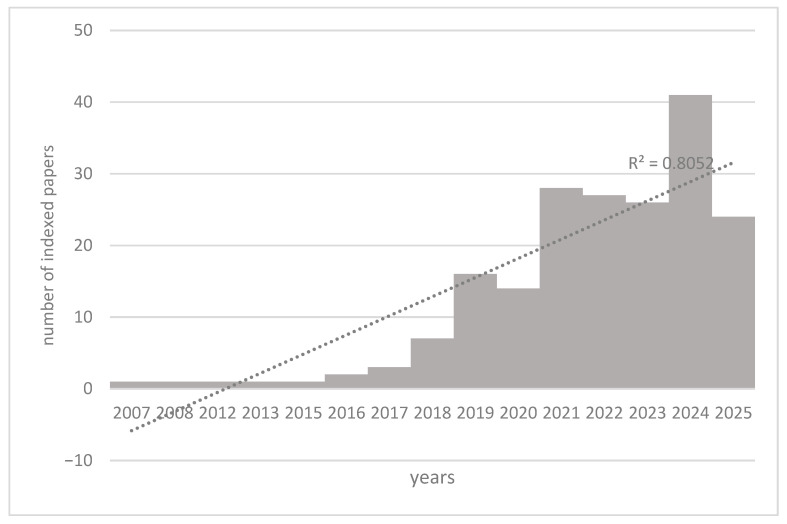
Trend of scientific articles indexed on PubMed from 2007 to early 2025 concerning Artificial Intelligence, Amyloid PET, and Alzheimer’s disease. The R^2^ value of 0.80 indicated that the linear model fits the data quite well, with a strong linear relationship between year and the number of publications.

As illustrated in Figure 3, the analysis revealed a clear trend of increasing interest and research output in the fields of Artificial Intelligence, Amyloid PET, and Alzheimer’s disease. The number of publications remained relatively low between 2007 and 2017, with a peak of three publications in 2017.

There has been a progressive increase starting in 2018 until reaching the peak in 2024. The increase in publications from 2018 to 2024 suggested growing research interest in Artificial Intelligence, Amyloid PET, and Alzheimer’s disease, even more stable than that previously evidenced for ^18^F-FDG PET (R^2^ = 0.80). This detailed breakdown highlights the dynamic nature of research in this interdisciplinary area.

Both ML and DL were applied in the analysis of Amyloid PET imaging with the aim to better classify Amyloid PET images into positive or negative with respect to visual assessment alone, and to obtain predictive data for evaluating the progression of MCI subjects into AD.

The ML algorithms were explored as a tool for the correct classification of Amyloid brain PET as positive or negative for Amyloid deposition, and the Random Forest test reached high values of sensitivity, specificity, and accuracy (86%, 92%, and 90%, respectively). Moreover, RF identified specific key regions of the brain for the correct PET classification [28].

Similar results were obtained using DL procedures. The CNN model showed 91% accuracy and 0.95 AUC, whereas visual assessment revealed positivity in 35.4% of the population and negativity in 64.6%. These findings suggested a high reliability of CNNs in distinguishing between positive and negative cases, reinforcing the idea that AI models might serve as a supplementary tool to enhance the accuracy of clinical diagnoses [29].

A longitudinal observational study evaluated the potential of radiomics features from Amyloid PET images as biomarkers of AD and predictors for evaluating the progression of MCI subjects [30]. The proposed AI model was able to distinguish AD patients from normal controls, with an AUC of 0.93, and to predict the development of AD from MCI with an AUC of 0.83.

Interesting results were also obtained with the correlation of the radiomic characteristics, clinical features, CSF data, and genetic alterations underlining the biological and clinical basis of radiomic data. These results are of great importance for the early clinical diagnosis or prediction of AD and MCI [30].

### 3.3. Artificial Intelligence, Tau PET, and Alzheimer’s Disease

Despite the more recent development of Tau tracers in Nuclear Medicine practice, a PubMed search for the keywords of “Artificial Intelligence”, “Tau PET”, and “Alzheimer’s disease” returned 81 selected articles, published in a relatively short period, between 2017 and 2025.

**Figure 4:**
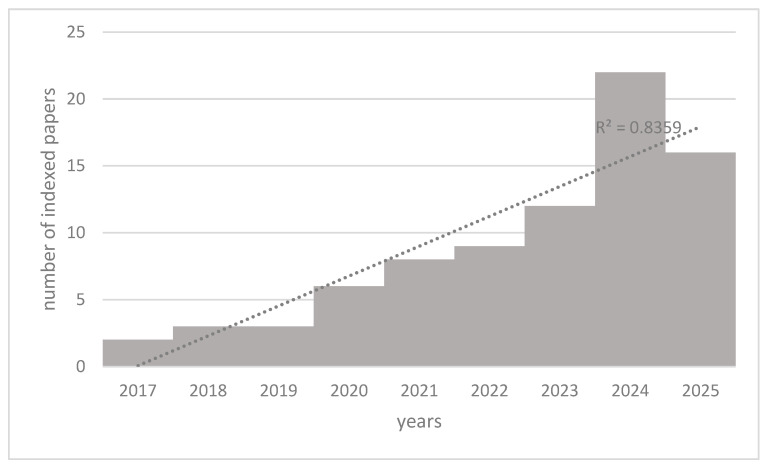
Trend of scientific articles indexed on PubMed from 2017 to early 2025 concerning Artificial Intelligence, Tau PET, and Alzheimer’s disease. The R^2^ value of 0.83 underlines a strong linear relationship between year and number of publications.

Figure 4 illustrates the trend of the published papers with a steadily increasing number of results over time (R^2^ = 0.83). The faster growth was observed in recent years, with a notable increase between 2022 and 2024, the latter with the highest number of results, indicating growing interest or research activity.

Advanced ML methods and radiomics models based on Tau PET results could be a useful support in clinical diagnosis, further providing evidence for identifying the risk factors in MCI patients. This model could be relevant in clinical practice since it accurately and consistently identified AD and MCI patients from normal controls with a high diagnostic accuracy (81.9 ± 6.1%) [31,32].

Multiple representative ML algorithms were compared for the evaluation of Tau PET imaging, showing that SVM was the most effective one for initial screening of AD/MCI vs. normal controls (accuracy of 96%). Its use in the evaluation of Tau deposition level is very effective in classifying AD stages, especially when PET data was associated with basal clinical information [33].

It was also evidenced that Deep Learning analysis could be useful to correlate Tau and Amyloid distribution, mainly in those cases with uncertain focal protein aggregates. In particular, CNNs were more useful with respect to standard analysis that correlated only with a widespread association between Tau PET and Amyloid PET images. CNNs better identified focal clusters of Tau accumulation in the bilateral medial temporal lobes, frontal lobes, precuneus, postcentral gyrus, and mid-cingulate that correlated with Amyloid PET [34]. The CNNs approach to Tau PET revealed new associations between Tau topography and Amyloid burden, thus contributing to better clarify the molecular pathogenesis of the extracellular Aβ-Amyloid instigation of intracellular Tau accumulation, which has been poorly understood until now [34].

Since Tau-PET is less accessible than other neuroimaging methods, it was investigated how Tau-PET images could be imputed (synthesized) from other image modalities such as T1-weighted MR (T1w), ^18^F-FDG PET, and Amyloid PET. They determined that a CNN model could impute Tau-PET images with high accuracy, with the best performance being achieved with the FDG-based model, followed by Amyloid PET and T1w MR [35].

## 4. Artificial Intelligence, Multimodal Data Integration, and Alzheimer’s Disease

To date, the application of AI to single diagnostic procedures has yielded promising results; however, this approach may fail to fully capture the complexity and heterogeneity inherent to AD.

In accordance with the AT(N) framework, several authors have proposed that AI could be effectively utilized to integrate data from multiple neuroimaging techniques alongside other sources such as fluid biomarkers, clinical information, and genetic analyses.

The integration of multimodal data has the potential to significantly enhance the accuracy and robustness of AI-based predictive models for AD by enabling the simultaneous evaluation of diverse pathological aspects of the disease [36]. This approach provided the opportunity for researchers to capture multiple aspects of the disease process, particularly in longitudinal studies [15,18].

Artificial Intelligence is able to integrate brain structural patterns provided by MR, brain metabolic assessment ascertained by ^18^F-FDG PET, and the presence of Amyloid or Tau clusters evidenced by Amyloid PET and Tau PET. Furthermore, the predictive capacity of AI models could be enhanced by adding data related to clinical information, cognitive and neuropsychological results, and genetic analysis regarding APOE ε4 allele status [23].

Machine Learning based on SVM analysis could manage the heterogeneous data obtained from ^18^F-FDG PET, MR, CSF biomarkers, and APOE genotype, showing high accuracy in the correct classification of AD cases and in predicting AD conversion in MCI subjects [15,37].

A multimodal DL framework based on the data obtained from MR, ^18^F-FDG PET, and neuropsychological tests showed high accuracy both in classifying AD (93%) cases and in predicting AD conversion (82%) [19].

AI models based on multimodality procedures showed higher performance compared with those based on the single-modality approach. In particular, RF classification improved AD classification when quantitative Amyloid PET and structural MR analyses were combined; the AUC values were 0.89 and 0.71 in differentiating AD versus normal controls and MCI, respectively [38].

In Table 2, we summarized the characteristics of the study populations (cohort size, data source, and external validation) of each primary reference.

## 5. Current Context, Future Directions, and Outstanding Challenges

The current literature indicates that AI applied to PET imaging can play a major role in the clinical management of AD, including the pre-processing phase of the data, which represents a crucial step, often necessary to obtain a reliable diagnostic performance [39]. The applications of AI analysis of PET imaging data in AD research have been extensive and multifaceted; the potential fields are different and may vary according to the clinical context, research focus, and/or the type of data being analyzed. Data derived from ^18^F-FDG PET, Amyloid PET, and Tau PET, which provided relevant insights into cerebral metabolism and pathological protein deposition, could be used for the early diagnosis of AD in high-risk subjects. Moreover, relevant improvement has been observed in the initial stages of the disease, particularly to predict MCI to AD progression, leveraging longitudinal PET studies to monitor the disease’s evolution and prognosis.

Furthermore, AI techniques proved to contribute to the identification and characterization of distinct AD subtypes, reflecting the heterogeneity of the disease, often by integrating PET biomarkers with other imaging modalities.

Crucially, the integration of multimodal data, combining PET neuroimaging findings with fluid biomarkers, comprehensive clinical information, and genetic profiles, has been leveraged to enhance diagnostic accuracy and support more informed clinical decision-making processes for patients with AD.

The substantial increase in the published literature reflects the growing integration and interdisciplinary collaboration among the fields of AI, PET imaging, and AD research. This positive trend suggests a potentially significant impact on clinical research and the development of innovative strategies for the management of AD. By fostering a deeper understanding of the disease and supporting more targeted diagnostic and therapeutic approaches, this convergence may contribute to improved outcomes in both research and clinical practice [17].

On the other hand, advances in AI automated methods, especially Machine Learning and Deep Learning, the technological and pharmaceutical developments in PET imaging, and the goal of “precision medicine”, which is increasingly taking on a decisive role [40], could drive further growth in publications.

The use of advanced AI algorithms such as Support Vector Machine, Convolutional Neural Network, Non-Convolutional Artificial Neural Network, Random Forest, and Logistic Regression improved PET imaging evaluation with greater accuracy than visual assessments alone [18,41].

Moreover, AI procedures have proven capable of integrating the data from different imaging technologies, CSF and serum biomarkers, and clinical information, thus providing an undeniable significant progress in AD diagnosis, prognosis, and predictive progression [42].

Notwithstanding the significant advancements and evident benefits that the application of AI in PET procedures holds for the management of AD, there are still hurdles that limit its widespread implementation in routine practice.

One potential issue is that AI models are often trained and tested on relatively homogeneous (e.g., single-center) datasets of limited sample size. Although selected casuistry and single-center studies for PET imaging may have the advantage of excluding potentially confounding factors, they may also fail to adequately represent the real variability of the AD population, thus weakening the effective performance of the AI algorithms that should ideally be validated on independent, large datasets [36].

To ensure access to broader and more representative case studies, variations in data quality, demographical differences, and variations in AD subtypes, various international datasets are being developed. The Global Alzheimer’s Association Interactive Network (GAAIN) is one of the most important and consolidated institutions that aims to collect independent Alzheimer’s disease databases worldwide by creating a global network of Alzheimer’s disease research centers able to share huge AD data [43].

The heterogeneity of ^18^F-FDG PET, Amyloid PET, and Tau PET data represents another limitation to the effective application of AI on PET imaging for the clinical management of AD. To guarantee the reliability of AI models as well as the reproducibility and comparability of the data, standardized acquisition and processing procedures are essential.

The Alzheimer’s Disease Neuroimaging Initiative (ADNI) and the Australian Imaging, Biomarkers and Lifestyle (AIBL) [44,45] are two of the most relevant associations that stimulate and recommend the use of standardized protocols in neuroimaging AD procedures in order to improve the congruence and the conformity of data for the AI dataset and to support collaboration between different researcher groups.

ADNI has been active since 2004, and the types of PET data collected have evolved over time, and it is still evolving. In the ADNI database for Amyloid PET scan, three radiopharmaceuticals were used for imaging: Florbetaben, Florbetapir, and NAV-4694. The targeted injected dose, the minimum injectable dose, and the imaging time after injection are also reported in a detailed table on the ADNI website (https://adni.loni.usc.edu/data-samples/adni-data/neuroimaging/pet/, accessed on 18 September 2025). It is also guaranteed that all ADNI scanners must be qualified through a process available in the PET Technical manual, thus not needing a re-qualification for the scanners unless there are substantial changes to hardware or software. Images, after undergoing pre-processing at the University of Michigan, are analyzed at UC Berkeley by means of an in-house software using native space, contemporaneous MR scans segmented and parcellated using FreeSurfer (Version 4.3), to be coregistered to the PET data, to provide PET measures in the Desikan–Killiany atlas. These data are available and include extensive methods documentation.

Furthermore, to preserve privacy and to avoid patient identification, ADNI images undergo de-/re-facing and multiple steps for quality control and data flow, as documented on the ADNI website. The ensemble of these processes guarantees the safe use and wide availability of data.

The Australian Imaging, Biomarker and Lifestyle (AIBL; https://aibl.org.au/research/, accessed on 18 September 2025) is an ongoing observational cohort study to highlight new insights into the onset and progression of Alzheimer’s disease, beginning in 2006 with an original cohort of 1112 recruited subjects over 60, and it is now the largest study of this kind in Australia, including more than 8500 person-years. Lifestyle, fluid markers, and neuroimaging are considered in AIBL.

Amyloid PET scans with 11C-PIB, Florbetaben, Florbetapir, Flutemetamol, and 18F-NAV46 94 of AD and MCI subjects have been included in AIBL since 2006.

Tau tracers (18F-MK6240) have been included since 2018; furthermore, MAO-B tracers (18F-SMBT-1) were considered to study astrogliosis to contribute to the knowledge of the pathophysiology of AD. Further details are available on the AIBL website.

Among its contributions, ADNI has recently introduced tools like the Berkeley PET Imaging Pipeline (B-PIP), which addresses multisite variability by normalizing image resolution and voxel size, enabling harmonized quantitative tracer uptake measurements across different scanners and tracers [46].

Moreover, it should be noted that the appropriate application of different PET procedures presupposes comprehensive knowledge of imaging protocols, awareness of tracer-specific limitations, and a critical understanding of the potential interpretive pitfalls associated with each PET modality in the evaluation of AD [47]. In particular, ^18^F-FDG PET imaging may be influenced by inter-site and scanner variability. Furthermore, a hypometabolic pattern may reflect comorbid conditions unrelated to AD, such as vascular diseases or other neurodegenerative disorders [47]. Amyloid PET positivity could also be observed in cognitively normal individuals, and the Amyloid deposition may reach a plateau in advanced stages, limiting its utility for the longitudinal assessment of disease progression [47]. The interpretation of Tau PET can be complicated by off-target binding (brainstem nuclei, substantia nigra, striatum, choroid plexus, leptomeninges, and blood vessels), and its clinical and research use is constrained by high cost and current limited accessibility [47].

An additional key factor for enabling full integration of AI tools into routine practice is the acceptance by clinicians and nuclear medicine physicians, who often see AI algorithms as complex and opaque, hence look at them suspiciously. Without a clear understanding of the mechanism behind an AI model, it becomes difficult both to ascertain when and/or when it may fail and to gauge its potential contribution to advancing the comprehension of the AD disorder [15,48].

For the acceptance of AI tools with a truly influential impact in clinical practice, it will be essential to create intuitive and straightforward interfaces that display the algorithmic results in a way that is both clinically significant and actionable [49].

To address this issue, the development and implementation of explainable AI (XAI) techniques have become increasingly important. Common XAI methods in neuroimaging include saliency maps, layer-wise relevance propagation (LRP), and SHAP (SHapley Additive exPlanations) values, which highlight the most influential features or brain regions contributing to AI model predictions. For instance, saliency maps can visualize which areas of PET imaging the model focuses on when classifying AD versus healthy controls, helping clinicians verify the biological plausibility of the AI’s decision. LRP provides pixel-wise relevance scores, making the decision process more interpretable, while SHAP values quantify the contribution of individual features, such as specific biomarkers or clinical variables, to the predictive outcome [50,51,52].

By incorporating these techniques, AI systems can offer transparent and clinically meaningful explanations, increasing clinician trust and facilitating the responsible integration of AI into diagnostic workflows. This interpretability not only supports clinical decision-making but also helps identify potential biases or errors in the models, contributing to safer and more effective applications in AD management.

In addition, beyond synthesizing existing evidence, it is important to align future research and reporting practices with established frameworks. Guidelines such as the Checklist for Artificial Intelligence in Medical Imaging (CLAIM) [53], the Transparent Reporting of a multivariable prediction model for Individual Prognosis or Diagnosis–AI extension (TRIPOD-AI), and the CONSORT-AI/SPIRIT-AI extensions provide structured recommendations for study design, reporting, and transparency in clinical AI research [54,55]. Incorporating these standards will facilitate reproducibility, improve methodological rigor, and support responsible translation of AI applications into clinical practice.

The implementation in the clinical use of AI models that provide real benefits to both patients and society also requires that AI models be developed and applied in an ethical and responsible manner. As a consequence, the application of AI models engenders critical ethical concerns, including issues pertaining to data privacy, confidentiality, security, informed consent, and transparency. It is also imperative to ensure that research participants are fully informed about both the benefits and potential risks related to the use of their data by other researchers, thereby safeguarding their rights and maintaining public trust [15].

Several authoritative frameworks and guidelines address these issues. For instance, the European Commission’s Ethics Guidelines for Trustworthy AI emphasize respect for human autonomy, prevention of harm, fairness, and explicability as core principles to guide AI development and deployment [56]. Similarly, the World Medical Association’s Declaration of Helsinki provides ethical standards for research involving human subjects, including aspects related to informed consent and data protection [57]. Additionally, the Health Insurance Portability and Accountability Act regulations in the United States establish legal requirements for safeguarding patient health information, critical for AI applications handling sensitive medical data [58].

Adherence to these frameworks is essential to ensure that AI integration in clinical practice is ethically sound, legally compliant, and socially acceptable.

## 6. Conclusions

Alzheimer’s disease is a progressive neurodegenerative disorder that involves social and economic fields and severely affects both patients and their families’ lives. Early diagnosis is crucial, yet often missed, as initial symptoms are easily mistaken for normal aging. Nuclear Medicine procedures have become essential to improve the accuracy of AD diagnosis, and PET imaging modalities provide unique and complementary information in the diagnosis and management of AD. The ^18^F-FDG PET imaging measures cerebral glucose metabolism, and Amyloid PET visualizes Amyloid-beta plaques and can identify abnormal deposition, even in preclinical stages. Furthermore, Tau PET imaging reveals the presence and distribution of Tau tangles, which are closely associated with disease progression and clinical symptoms.

While PET imaging yields valuable molecular and metabolic biomarkers, the complexity and volume of imaging data pose significant challenges for conventional analytic methods. AI models, particularly ML and DL algorithms, offer transformative potential in enhancing the diagnostic utility of PET.

AI model frameworks can efficiently process and integrate multi-tracer PET data, improving sensitivity and specificity in early AD detection. In this context, ML and DL trained on ^18^F-FDG PET, Amyloid PET, and Tau PET imaging have shown promise in early diagnosis of AD, in predicting MCI conversion, in tracking disease progression, and in predicting cognitive decline trajectories.

Moreover, to enhance diagnostic precision, the integration of different data has gained attention. AI procedures could process contemporary PET imaging, MR technique, clinical information, serum and CSF biomarkers, and genetic patient data. This approach offers a more comprehensive view of the brain by integrating anatomical, functional, and molecular data.

When applied to PET and multimodal imaging, AI not only enhances image analysis but also facilitates cross-modality integration, improving both diagnostic accuracy and efficiency.

Despite these promising developments, several challenges remain. These include a shortage of large, high-quality, and standardized datasets to train robust AI models; the limited generalizability across populations and imaging devices; and concerns over data privacy, clinical validation, and interpretability. Moreover, resistance from some areas of the medical community still exists, partly due to concerns over trust, liability, and the ethical implications of relying on automated systems in clinical decision-making.

In conclusion, the application of Artificial Intelligence to PET and multimodal neuroimaging constitutes a significant advancement in the early and accurate diagnosis of Alzheimer’s disease. Despite the promising results demonstrated thus far, the successful translation of these technologies into routine clinical practice will require addressing critical challenges, including data standardization, multicenter validation, algorithmic transparency, and adequate training of healthcare professionals. The establishment of collaborative and interdisciplinary approaches will be essential to facilitate this transition. In the absence of curative therapies, AI-driven tools have the potential to substantially improve disease management by enabling earlier detection, more personalized assessments, and, ultimately, better clinical outcomes and quality of life for patients and their caregivers.

## Figures and Tables

**Table 1:** Summary of AI procedures applied to PET and PET/CT neuroimaging and suggested main purposes in AD.

AI Procedures	Representative References	Main Purpose
**Machine Learning** Supervised Learning (SVM *, Logistic Regression, Decision Trees, and K-Nearest Neighbors) Ensemble Learning (Bagging, Boosting, and Random Forests)	[15,18,19,20,21]	AD classification MCI/AD conversion prediction AD subtype identification Improving the performance of Primary classifiers
**Deep Learning** (CNNs **, RNNs ***, Autoencoder)	[18,22]	AD classification MCI/AD conversion prediction AD progression prediction Features learning and extraction
**Combined Methods** (DL Features Extraction + ML Classification; Unsupervised Learning + ML Classification)	[23,24]	AD classification AD subtype identification Multimodal data integration

* SVM = Support Vector Machine; ** CNNs = Convolutional Neural Networks; *** RNNs = Recurrent Neural Networks.

**Table 2:** Characteristics of the study populations. Key to acronyms/abbreviations: AddNeuroMed = The European Collaboration for the Discovery of Novel Biomarkers for Alzheimer’s Disease, ADNI = Alzheimer’s Disease Neuroimaging Initiative, AIBL = Australian Imaging Biomarkers and Lifestyle Study of Aging, GAAIN = The Global Alzheimer’s Association Interactive Network.

	Cohort Size	Data Source	External Validation
Zhang et al. [19]	202	ADNI	No
Lebedev et al. [20]	896	ADNI + AddNeuroMed	Yes
Kishore & Goel [22]	83	ADNI	No
Nuvoli et al. [25]	150	Single-center	No
Ding et al. [26]	1042	ADNI + Single center	Yes
Ryoo et al. [27]	1607	ADNI	No
Zukotynski et al. [28]	57	Multicenter	No
An et al. [29]	175	Single-center (?)	No
Ding et al. [30]	1078	ADNI	No
Jiao et al. [31]	642	ADNI + Single-center	Yes
Park et al. [33]	199	ADNI	No
Gupta et al. [37]	158	ADNI	No
Bao et al. [38]	261	AIBL + GAAIN + Single-center	Yes

## Data Availability

No new data were created or analyzed in this study.

## Abbreviations

The following abbreviations are used in this manuscript:

AI	Artificial Intelligence
AD	Alzheimer’s Disease
PET	Positron Emission Tomography
MCI	Mild Cognitive Impairment
SCD	Subjective Cognitive Decline
CSF	CerebroSpinal Fluid
ML	Machine Learning
DL	Deep Learning
SVM	Support Vector Machine
RF	Random Forest
CNNs	Convolutional Neural Networks
RNNs	Recurrent Neural Networks
GAAIN	Global Alzheimer’s Association Interactive Network
ADNI	Alzheimer’s Disease Neuroimaging Initiative
AIBL	Australian Imaging, Biomarkers and Lifestyle
XAI	Explainable AI
